# Radiation cystitis modeling: A comparative study of bladder fibrosis radio‐sensitivity in C57BL/6, C3H, and BALB/c mice

**DOI:** 10.14814/phy2.14377

**Published:** 2020-02-28

**Authors:** Bernadette M. M. Zwaans, Kyle A. Wegner, Sarah N. Bartolone, Chad M. Vezina, Michael B. Chancellor, Laura E. Lamb

**Affiliations:** ^1^ Department of Urology William Beaumont Hospital Royal Oak MI USA; ^2^ Oakland University William Beaumont School of Medicine Royal Oak MI USA; ^3^ Molecular and Environmental Toxicology Center School of Medicine and Public Health University of Wisconsin‐Madison Madison WI USA; ^4^ Department of Comparative Biosciences School of Veterinary Medicine University of Wisconsin‐Madison Madison WI USA

**Keywords:** Collagen, Fibrosis, Frequency, Radiation Cystitis

## Abstract

A subset of patients receiving radiation therapy for pelvic cancer develop radiation cystitis, a complication characterized by mucosal cell death, inflammation, hematuria, and bladder fibrosis. Radiation cystitis can reduce bladder capacity, cause incontinence, and impair voiding function so severely that patients require surgical intervention. Factors influencing onset and severity of radiation cystitis are not fully known. We tested the hypothesis that genetic background is a contributing factor. We irradiated bladders of female C57BL/6, C3H, and BALB/c mice and evaluated urinary voiding function, bladder shape, histology, collagen composition, and distribution of collagen‐producing cells. We found that the genetic background profoundly affects the severity of radiation‐induced bladder fibrosis and urinary voiding dysfunction. C57BL/6 mice are most susceptible and C3H mice are most resistant. Irradiated C57BL/6 mouse bladders are misshapen and express more abundant collagen I and III proteins than irradiated C3H and BALB/c bladders. We localized *Col1a1* and *Col3a1* mRNAs to FSP1‐negative stromal cells in the bladder lamina propria and detrusor. The number of collagen I and collagen III‐producing cells can predict the average voided volume of a mouse. Collectively, we show that genetic factors confer sensitivity to radiation cystitis, establish C57BL/6 mice as a sensitive preclinical model, and identify a potential role for FSP1‐negative stromal cells in radiation‐induced bladder fibrosis.

## Graphical abstract

Radiation cystitis, a severely debilitating condition characterized by fibrosis, vascular damage, and compromised bladder function, develops in cancer survivors who received pelvic radiation treatment. This is the first study to compare the effects of radiation on the bladder across different murine genetic backgrounds, which may explain differences in patients’ sensitivity to developing radiation cystitis after radiation cancer treatment.

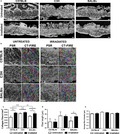

## 1 INTRODUCTION

Radiation therapy is used to treat a variety of pelvic cancers, including prostate and cervical cancer (Society AC, 2016). While radiation can be very effective, it can also damage benign tissue in the radiation field, causing long‐term side effects. Radiation‐induced bladder damage can result in a condition called radiation cystitis (RC, also known as hemorrhagic cystitis). The reported incidence of RC ranges from 23%–80% due to the variability in types and doses of radiation (Browne et al., 2015). A recent study reported an incidence of RC of 10% in prostate cancer survivors that had received radiation therapy (Martin et al., 2019). The incidence of RC is higher in men due to the frequent use of radiation therapy to treat prostate cancer (Browne et al., 2015). However, there is no evidence of increased bladder radio‐sensitivity in males versus females. The variation in susceptibility to developing RC after treatment may also be explained in part by genetic variability among patients. RC progresses in three phases: an acute phase continuing for several weeks after radiation therapy, an asymptomatic latent phase, and a late phase that can start as early as 6 months or as late as 15 years after radiation treatment (Zwaans, Chancellor, & Lamb, 2015). Patients with RC suffer from progressive lower urinary tract symptoms, which may include increased urinary frequency, nocturia, urinary incontinence, pelvic pain, and hematuria. Hematuria can range from microscopic to macroscopic with blood clots that require blood transfusions. A patient's risk of developing RC increases with radiation dose and number of radiation fractions but these factors alone do not predict which patients will develop RC (Zwaans et al., 2018). Thus, there is a tremendous need for new and reliable RC therapies, biomarkers to predict patient susceptibility and to predict patient candidacy for early intervention.

The lack of reliable and safe RC treatment options is in part due to the limited knowledge of how RC develops. One major culprit is the progressive buildup of fibrotic tissue, a process that is addressed by existing therapies. Bladder fibrosis has an inverse relationship with bladder compliance, and patients with a fibrotic bladder suffer from lower urinary tract symptoms such as increased urinary frequency and nocturia (Fry et al., 2018). Fibrosis is caused by an imbalance between extracellular matrix production and breakdown. Excessive accumulation of extracellular matrix proteins, such as collagens and fibronectin, interferes with normal organ function (Herrera, Henke, & Bitterman, 2018). Chronic inflammation and subsequent tissue damage is one trigger for tissue fibrosis. Factors that give rise to chronic bladder inflammation include radiation therapy (e.g., RC), chemical exposure (e.g., cyclophosphamide or ketamine cystitis), old age, diabetes, genetics, or through unknown causes (e.g., interstitial cystitis) (Droller, Saral, & Santos, 1982; Gharaee‐Kermani et al., 2013; Ikeda et al., 2018; Wang et al., 2017; Zwaans et al., 2016).

Several laboratories are developing preclinical models to identify pathways involved in RC and its fibrotic sequelae with the goal of formulating novel therapeutics (Ikeda et al., 2018; Jaal & Dorr, 2006; Zwaans et al., 2016). Many laboratories use mice as experimental models, but no study has compared bladder radio‐sensitivity across mouse strains. Different mouse strains can be used for exploring genetic variability with respect to human biology. Susceptibility to radiation‐induced damage of the lung and other organ systems is strain and organ dependent (Ao et al., 2009; Kalash et al., 2014; Walkin et al., 2013). Mice with a strong C57BL/6 background are more susceptible to radiation‐induced pulmonary and intestinal fibrosis while mice with a C3H background are resistant (Franko, Sharplin, Ward, & Taylor, 1996; Iwakawa et al., 2004; Johnston, Piedboeuf, Baggs, Rubin, & Finkelstein, 1995; Skwarchuk & Travis, 1998). However, C57BL/6 mice are not always highly susceptible to pro‐fibrotic stimuli (Walkin et al., 2013). For example, C57BL/6 mice are resistant to diabetes‐induced renal fibrosis, but prone to urinary outlet obstruction‐induced renal fibrosis (Puri et al., 2010; Sugimoto, Grahovac, Zeisberg, & Kalluri, 2007).

In this study, we compared anatomical, histological, and physiological responses to bladder irradiation in females across three mouse strains: C57BL/6, C3H, and BALB/c. The rank order sensitivity to radiation‐induced bladder fibrosis is: C57BL/6 > BALB/c > C3H. We also identified FSP1‐negative stromal cells as putative origins of radiation‐induced bladder fibrosis.

## 2 MATERIALS AND METHODS

### 2.1 Animals

All experimental procedures were reviewed and approved by Beaumont Health's IACUC (AL‐16–02). Female C57BL/6 (strain code 027), C3H (strain code 025), and BALB/c mice (strain code 028) (Charles River) were housed under standard housing conditions with 4–5 mice per cage, fed a soy protein‐free extrudent rodent diet, and received clean cages weekly. Female mice were chosen as they are much easier to catheterize than male mice. Mice were 6 weeks of age at baseline with an average body weight of 14.75 g and 14.83 g (C57BL/6), 16.5 g and 16.67 g (C3H), and 16.25 g and 16.5 g (BALB/c) for the untreated and irradiated groups, respectively.

### 2.2 Radiation treatment

Radiation was performed on the SARRP unit (Xstrahl Life Sciences) as previously described.( Zwaans et al., 2016) In brief, 6‐week‐old C57BL/6, C3H, and BALB/c mice were anesthetized with 2%–3% isoflurane and maintained on 1.5%–2.0% isoflurane throughout the procedure. Mice were positioned on the SARRP mouse platform, a computerized tomography (CT) was taken and the target for irradiation set in the middle of the bladder. The appropriate SARRP collimator (3x3 mm or 5x5 mm) was installed and a total of 40 Gy at a rate of 2 Gy/min was delivered through two separate beams, positioned at opposing angles, to minimize damage to surrounding tissue. The size of the collimator was determined based on the size of the bladder at the onset of treatment to maximize bladder coverage. The beam settings were 220 kV and 13 mA, delivered through a 0.15 mm Cu filter for treatment. Control animals were anesthetized for the same duration as irradiated mice but did not receive radiation. Imaging and irradiation for each animal was accomplished within 30–60 min. Following successful irradiation, mice recovered in a warming tank, and returned to general housing.

### 2.3 Micturition

Mouse urinary function was measured biweekly using the void spot assay as previously described (Wegner et al., 2018). Mice were housed singly for 4 hr (10a.m. – 2p.m. ± 5 min) in a standard sized cage lined with filter paper (Cat# 1,650,921; BioRad, Hercules, CA) with access to food but not water. Minimal enrichments were added to each cage to minimize chewing of filter paper (rounded house with open bottom and small piece of string paper). After 4 hr, filter paper was removed and imaged under UV light. The total number of spots, spot size, total void volume, and primary void size were measured using ImageJ software and the monthly data points were averages of 2–3 micturition experiments performed in 1 month.

### 2.4 Tissue harvest

Animals were sacrificed 12 months post‐irradiation. Mice were first anesthetized, catheterized, and instilled with 100–120 µl sterile saline. The bladders were exposed through a small abdominal incision and bladders were photographed. Bladders were subsequently dissected, cut into two strips, fixed in 4% paraformaldehyde for 24 hr at 4°C, and processed and embedded in paraffin blocks.

### 2.5 Masson's Trichrome staining

Bladder sections (4 µm) were stained with Masson's Trichrome to detect the presence of collagen (TRM‐2‐IFU, SCYTEK Laboratories, Logan, UT). All slides were warmed to 60°C overnight and subjected to a series of deparaffinization and rehydration steps. Trichrome staining was performed according to the manufacturer's instructions. The amount of fibrosis (blue stain) was quantified using ImageJ software using the color deconvolution plugin tool. For each bladder strip, the percentage of tissue positive for fibrosis was measured in a minimum of eight regions of interests (ROI’s, 100 x 100 µm). These ROI’s encompassed tissue from the detrusor region of the bladder strip. The average percentage of fibrosis in the measured ROI’s was calculated per bladder tissue, and subsequently the averages per treatment group (*n* = 3–6 bladders) and per strain displayed.

### 2.6 Immunofluorescent staining and analysis for collagen

For immunofluorescence staining of paraffin sections, antigen retrieval was performed in sodium citrate buffer, pH 6.0. Slides were blocked in 5% goat serum, 1% BSA, and 1% Roche Blocking Reagent (Cat#50‐100–3304; Roche‐Blocking Reagent, Fisher Scientific, Waltham, MA) in TBS with 0.1% tween20 (TBSTw). Primary antibody was incubated in blocking buffer overnight at 4°C. Primary antibodies used for single staining were Collagen I (1:100; Novus Biologicals, NB600‐450, RRID:AB_522923) and Collagen III (1:100; Novus Biologicals, NB600‐594, RRID:AB_10001330). Slides were washed in TBSTw and incubated with goat anti‐rabbit Alexa Fluor 488 (1:250; Thermo Fisher Scientific Cat# A11070; RRID:AB_2534114) for 60 min at RT. Tissue sections were washed again in TBSTw, counterstained with DAPI (4′,6‐diamidino‐2‐phenylindole) and mounted in ProlonGold.

Fluorescent images were captured on an Olympus BX43 microscope at 40x magnification. A minimum of five images per bladder tissue were taken and, per image, the amount of fluorescence, based on a minimum fluorescence threshold, in two regions of interests (ROI’s, 100 x 100 µm) were measured using ImageJ software. These ROI’s encompassed tissue from the detrusor region of the bladder strip. The average percentage of tissue with positive fluorescent staining of collagen I or III was calculated per bladder, and subsequently the averages per treatment group (*n* = 3–6 bladders) and per strain displayed.

### 2.7 Picrosirius Red (PSR) staining and analysis

Bladder strip sections were deparaffinized and rehydrated through a graded ethanol series. Slides were incubated for 1 hr at 25°C in PSR staining solution [0.5 grams of Direct Red 80 (2610– 10–8; Sigma‐Aldrich Corp.) in 500 ml of saturated picric acid (P6744‐ 1GA; Sigma‐Aldrich Corp., St. Louis, MO). Slides were washed twice with acidified water (0.5% Acetic Acid), dehydrated in graded ethanols, cleared in xylene, and mounted using Richard–Allan toluene‐based mounting medium (4112APG; Thermo Fisher Scientific).

Imaging was performed using a Leica SP8 Confocal Microscope (Leica, Wetzlar, Germany) using a 20x oil immersion objective (HC PL Apo CS2 NA = 0.75; Leica, Wetzlar, Germany). PSR staining was visualized by exciting the samples using a 561 nm laser and detecting emission between 635–685 nm. Autofluorescence of the tissue was visualized by exciting with a 405 nm laser and detecting emission between 480–525 nm. Images were captured at 1024 x 1024 resolution using LAS X software (Leica, Wetzlar, Germany). Tile scanning was used to stitch images and generate an image across the entire bladder strip.

To eliminate non‐collagenous autofluorescent features, including red blood cells and elastin fibers from analysis, FIJI image software (LOCI, Madison, WI) was used to digitally subtract the autofluorescent image from the PSR image, leaving only true PSR staining for quantification.

To ensure uniform quantification, 0.2 mm^2^ ROI’s were selected from the center of each bladder strip. These ROI’s encompassed tissue from both the lamina propria and detrusor regions of the bladder strip. ROI images were converted to 8‐bit, 512 x 512 resolution image files. Total collagen density was determined by quantifying the area of the image occupied by PSR‐stained collagen relative to the entire area of the ROI. Individual collagen fiber metrics including density, length, and diameter were measured using CT‐FIRE fiber detection software (Bredfeldt et al., 2014) (LOCI, Madison, WI). Three ROI’s were analyzed from *n* = 3–6 mice per treatment group. Mean collagen metrics were calculated for each ROI.

### 2.8 RNAScope analysis and fibroblast immunostaining

RNAScope‐mediated RNA detection was performed as described by the manufacturer (Cat # 322360, Advanced Cell Diagnostics). In short, slides were warmed at 60°C overnight, deparaffinized, peroxide activity blocked, antigen retrieval performed, and treated with Protease Plus for 7.5 min. Subsequently, bladder sections were incubated with RNA probes specific to collagen I (Mm‐Col1a1, Cat# 319371, Advanced Cell Diagnostics) and collagen III (Mm‐Col3a1, Cat# 455771, Advanced Cell Diagnostics) for 2 hr at 40°C. Subsequently, tissue sections underwent a series of amplification steps at 40°C before signal development. Sections were counterstained with methyl green (H‐3402, Vector Labs, Burlingame, CA), washed in distilled water and covered with a cover slip for imaging. After imaging, coverslips were carefully removed, tissue sections were refixed in 10% PFA for 10 min at RT, washed in TBSTw, and blocked in in 5% goat serum, 1% BSA, and 1% Roche Blocking Reagent (Cat#50‐100–3304; Roche‐Blocking Reagent, Fisher Scientific, Waltham, MA) in TBSTw. Sections were incubated with Fibroblast Specific Protein 1 (FSP‐1, also known as S100A4; 1:100; Abcam ab41532, RRID:AB_945346) overnight at 4°C with gentle agitation, washed in TBSTw, incubated with goat anti‐rabbit HRP‐conjugated IgG (MP‐7451, Vector Labs, Burlingame, CA), and developed using DAB (SK4105, Vector Labs).

Images were captured on an Olympus BX43 microscope at 20x magnification. A minimum of three images were captured of the RNA scope only staining, and of each image the number of collagen I or III positive cells were counted in 2–3 ROI’s (200 x 200 µm) using ImageJ software. These ROI’s encompassed tissue from the detrusor region of the bladder strip. The average number of collagen I or III‐positive cells per bladder were calculated, and the averages per treatment group (*n* = 3) and per strain displayed.

### 2.9 Statistical analysis

All statistical analyses were carried out using SPSS 26. Micturition measurements were analyzed using a multiple t‐test assuming equal variances in the data with Bonferroni correction. For trichrome, PSR, collagen, and RNAscope analysis (Figures 3, 4, 5, 6), a Levene's test was used to test whether homogeneity of variances was the same between groups. Statistical significance was determined by a univariate two‐way ANOVA, with the fixed effects being treatment, strain, and interaction with a random intercept for mouse, followed by a Bonferroni post hoc test. A nonparametric Student's t‐test assuming equal variance in the data with Bonferroni correction was performed to analyze the effect of radiation treatment alone within each strain. Multiple linear regression analysis was carried out to determine if collagen I and collagen III expression could predict radiation‐induced fibrosis in the bladder. For all statistical analysis, a difference of *p* < .05 was considered significant.

## 3 RESULTS

### 3.1 Bladder radiation causes a persistent increase in micturition frequency

The bladder response to radiation was compared across C57BL/6, C3H, and BALB/c mice. These strains are commonly used in biomedical research and differ in sensitivity to lung and interstitial cell radiation damage (Walkin et al., 2013). The percent of bladder receiving the full 40 Gy radiation dose was 68.5% (range: 51.3%–91%). A single bladder exposure of 40 Gy radiation is well‐tolerated by all mouse strains as no change in bodyweight is observed, nor do the mice develop any apparent skin lesions because of the treatment in the weeks post‐radiation. A statistically significant lower bodyweight is observed in irradiated C3H mice in comparison to littermate controls starting at 20 weeks post‐radiation (Figure S1a). This has not previously been observed by us or other groups in C3H mice after irradiation (Zwaans et al., 2016). By 12 weeks post‐radiation, graying of the fur, or minor hair loss appears at the site of entry and exit beam in C57BL/6 and C3H mice, respectively, and remains visible until the end of the study (Figure S1b). BALB/c mice experience no changes in coat color or hair loss after radiation (Figure S1b).

**Figure 1:**
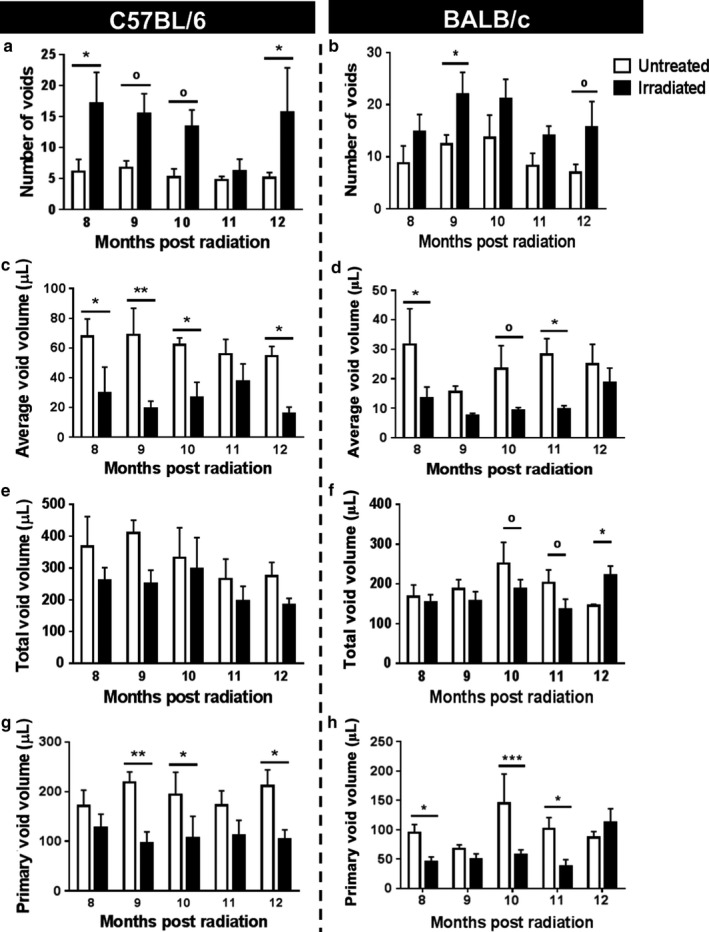
Radiation increases micturition frequency and reduces average volume per void in C57BL/6 and BALB/c mice*. *Micturition patterns were measured of C57BL/6 (a–d) and BALB/c (e–h) mice in response to irradiation. Micturition patterns were analyzed for total number of voids (a and e), average void size (b and f), total voided volume (c and g), and volume of primary void (d and h). C57BL/6: Untreated *n* = 4, Irradiated *n* = 4 (8–9 months), and *n* = 3 (10–12 months); BALB/c: Untreated *n* = 4, Irradiated *n* = 6 (8 months), and *n* = 5 (9–12 months). Error bars = *SD*. ^o^ 0.05 < *p* ≤ .08, * *p* < .05, ** *p* < .01, *** *p* < .001

The impact of bladder radiation on mouse micturition patterns was evaluated using the void spot assay, which singly houses mice for 4 hr on cages lined with filter paper to capture the pattern of spontaneous voiding (Wegner et al., 2018). C3H mice are destructive to the filter paper and thus their micturition patterns cannot be analyzed using this method. Radiation increases the number of voids recorded over a 4 hr period (Figure 1a,b) and reduced average void volume (Figure 1c,d) without affecting total voided volume (Figure 1e,f) in C57BL/6 and BALB/c mice. Radiation reduced the volume of the primary void, the largest detected void on the paper (Figure 1g,h). Overall, both C57BL/6 and BALB/c mice show radiation‐induced micturition changes; however, the effect is more pronounced and detected earlier in C57BL/6 mice.

### 3.2 Radiation changes bladder shape and causes bladder fibrosis

**Figure 2:**
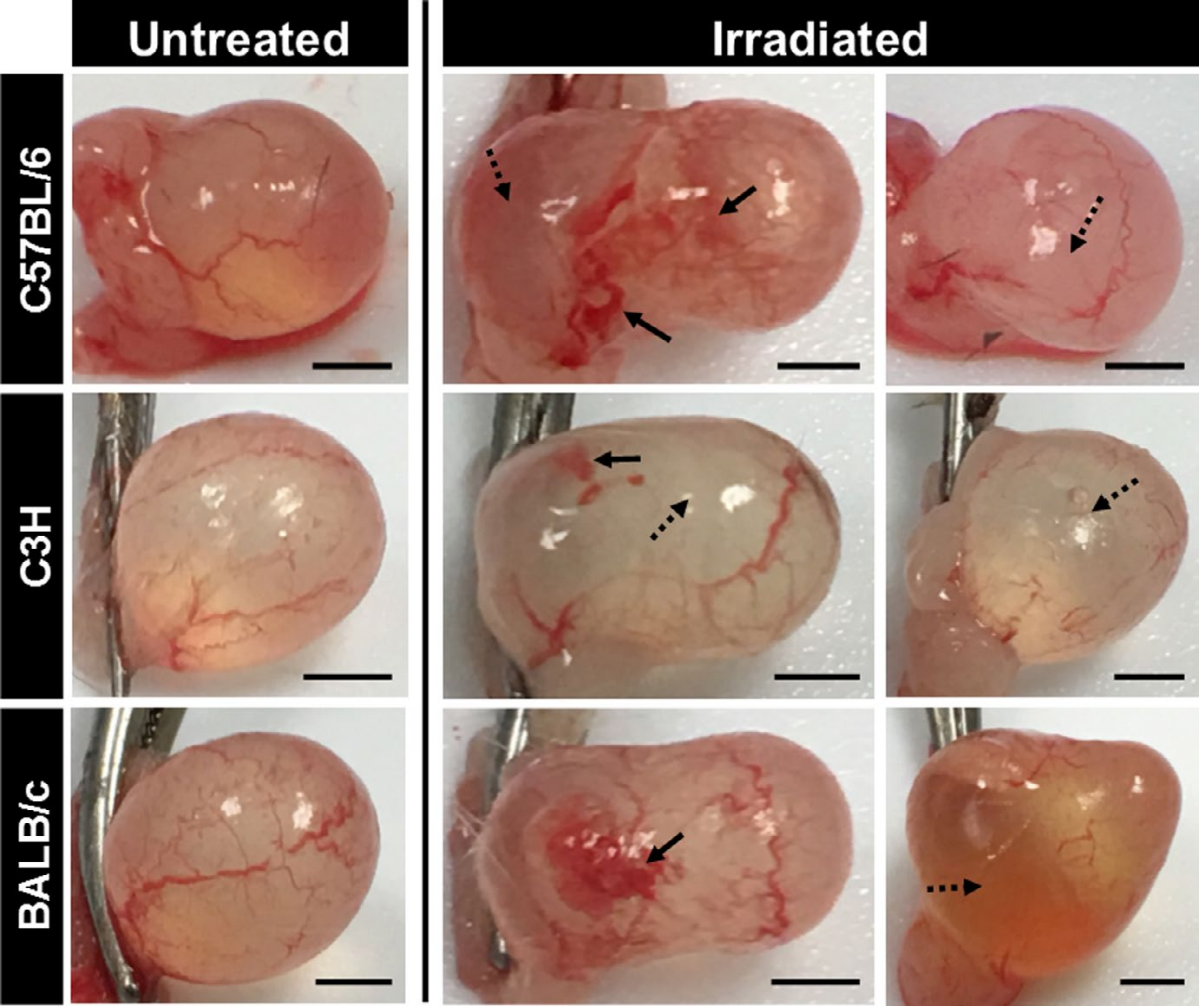
Radiation exposure induces a long‐lasting change in bladder shape*. *Macroscopic images of freshly harvested bladder tissues from three different mouse strains are shown at 12 months post‐radiation and compared to untreated littermate controls. Bladder deformation is detected in all irradiated bladders, while untreated bladders maintain a rounded shape. Full arrows: areas of vascular damage (hemorrhaging or telangiectasia); stripped arrows: avascular areas. Black scale bar = 2 mm

**Figure 3:**
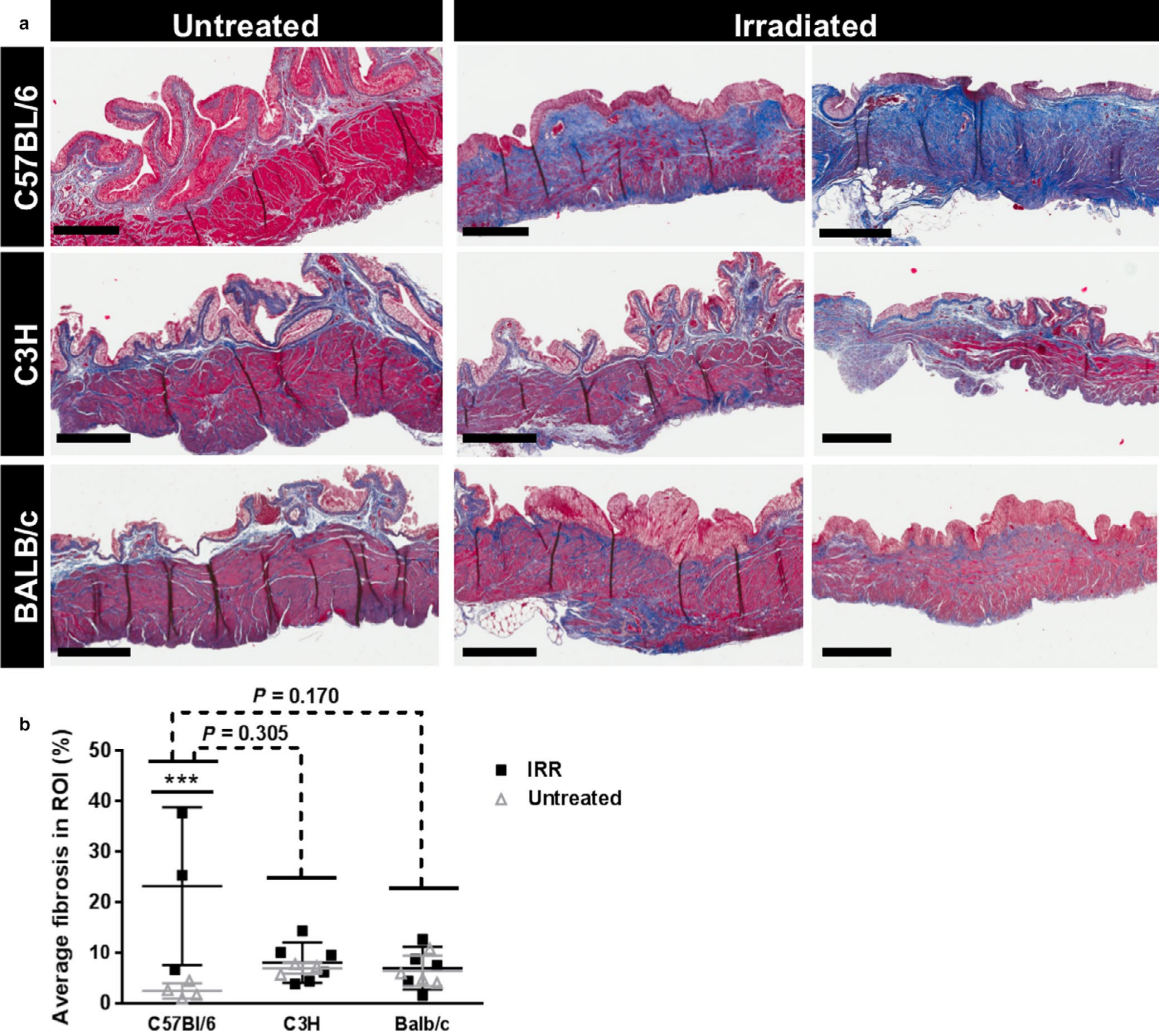
Radiation‐induced fibrosis in mouse bladder is strain‐dependent*. *Masson's trichrome staining was performed on untreated and irradiated mouse bladder strips for all three mouse strains. Increased fibrosis is detected in the C57BL/6 mice in response to radiation. Results are mean ± *SD* of *n* = 3–6 mice. Dashed line: ANOVA; Full line: multiple t‐test. *** *p* < .001. Scale bar = 400 µm

Mouse bladders were harvested 12 months post‐radiation. Radiation deforms bladder shape in all three mouse strains, an effect persisting at least 12 months after the single radiation dose (Figure 2). Pale avascular lesions (Figure 2, dotted arrows) hemorrhage, and telangiectasia are observed in irradiated but not in control bladders (Figure 2, full arrows). Masson's Trichrome was used to stain the extracellular matrix blue (Figure 3a) and the amount of blue staining was used as a proxy for fibrosis present in the tissue. ANOVA revealed a significant interaction between radiation treatment and mouse strain (Two‐way ANOVA, *p* = .006, partial η^2^ = 0.418) in the development of fibrosis as measured with Masson's trichrome stain. However, the Bonferroni post hoc test failed to identify statistical significance between strains. Analysis of the effect of radiation within each strain demonstrated a significant increase in bladder fibrosis in response to radiation in C57BL/6 mice (*p* < .001), but not in C3H or BALB/c mice (Figure 3b).

### 3.3 Bladder radiation causes increased bladder collagen fiber density in a mouse strain‐dependent manner

**Figure 4:**
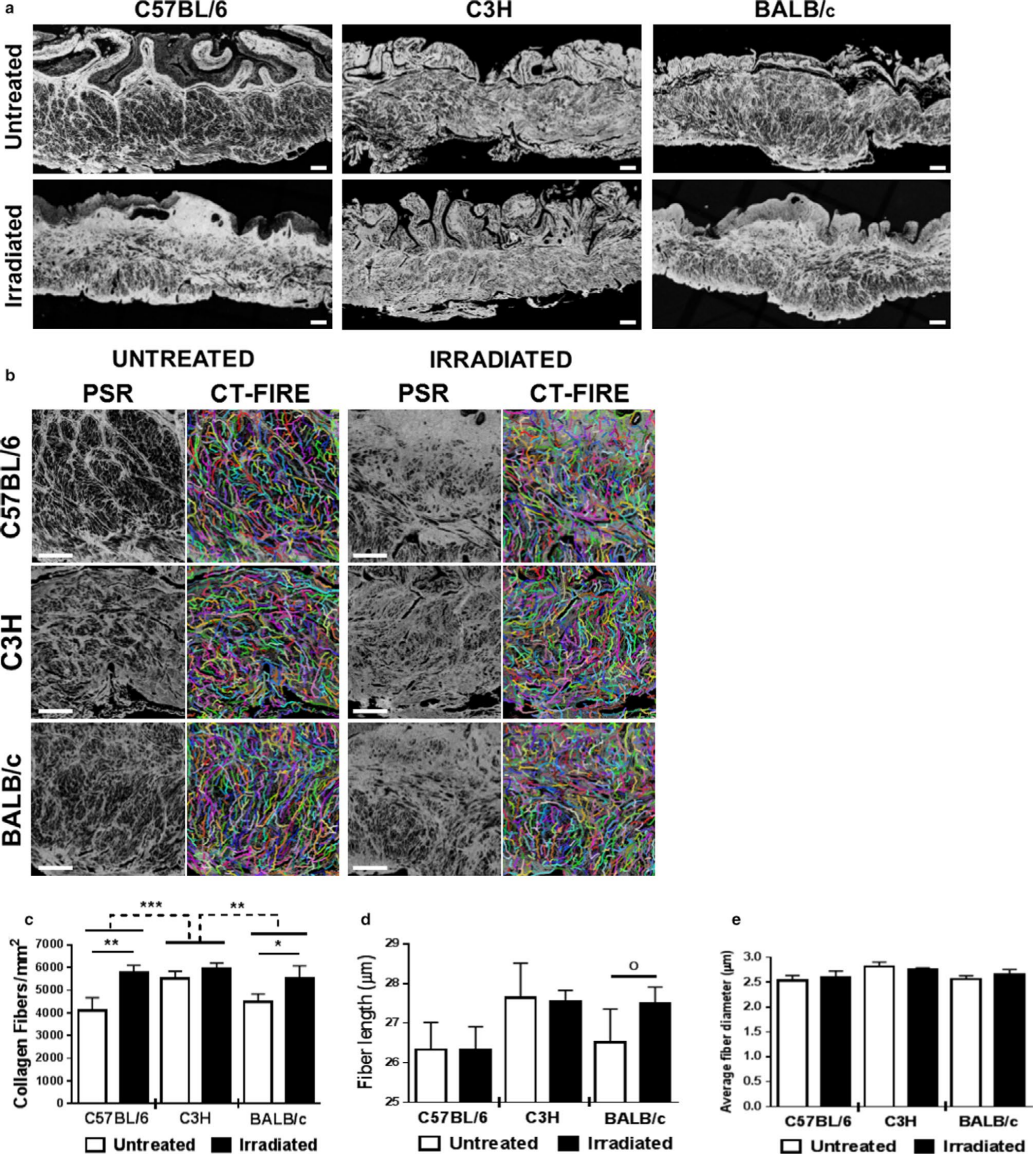
Increased collagen deposition and altered fiber metrics associated with radiation*.* Bladder strips from irradiated and control mice were stained with picrosirius red and imaged using fluorescent microscopy. (a) Irradiation triggers collagen accumulation in both C57BL/6 and BALB/c mouse strains. (b) CT‐FIRE was used to quantify collagen fiber abundance, length, and diameter in 0.2 mm^2^ regions of interest (ROI). (c–e) Irradiation triggers fibrotic shifts in collagen architecture of C57BL/6 and BALB/c mouse strains. Both C57BL/6 and BALB/c mouse strains had a significant increase in fibrillar collagen density within the bladder wall (c); however, only BALB/c mice have significant increases in average length (d) and diameter (e) of fibers. C3H mice experience no overall changes in average fiber abundance, length, or diameter. Results are mean ± *SD* of *n* = 3–6 mice. Dashed line: ANOVA; Full line: multiple t‐test. ^o^ *p* ≤ .08, *p < .05, **p < .01, ***p < .001. Scale bar = 100 µm

Collagen is an extracellular matrix protein known to play a role in fibrosis. Collagen density was measured by staining bladder tissue sections with picrosirius red and imaging the stained sections under fluorescent light, a method shown previously to detect fibrillar and nonfibrillar collagens (Wegner, Keikhosravi, Eliceiri, & Vezina, 2017). Radiation significantly increases collagen fiber density in the nonepithelial portions of the C57BL/6 and BALB/c mouse bladder wall (Figure 4a). C3H strain mice appear to be resistant to radiation‐mediated changes in bladder collagen fiber density.

Density, length, and diameter of individual collagen fibers were quantified using CT‐FIRE fiber detection software (Figure 4b‐c). The Levene's test demonstrates homogeneity within the data. The effect of radiation treatment on collagen fiber density, measured by fluorescent imaging of PSR staining, is impacted by the fixed variable “strain” (ANOVA *p* = .038, partial η^2^ = 0.32). The Bonferroni post hoc test identifies a significant difference between C57BL/6 and C3H mice (*p* = .001) and between BALB/c and C3H mice (*p* = .022). In addition, radiation significantly increases the number of collagen fibers per mm^2^ in C57BL/6 and BALB/c mouse but not in C3H mice. Strain did not influence the effect of radiation on fiber length or fiber diameter (Figure 4d,e).

### 3.4 Collagen I is more abundant in fibrosis‐prone C57BL/6 mice 12 months after radiation

**Figure 5:**
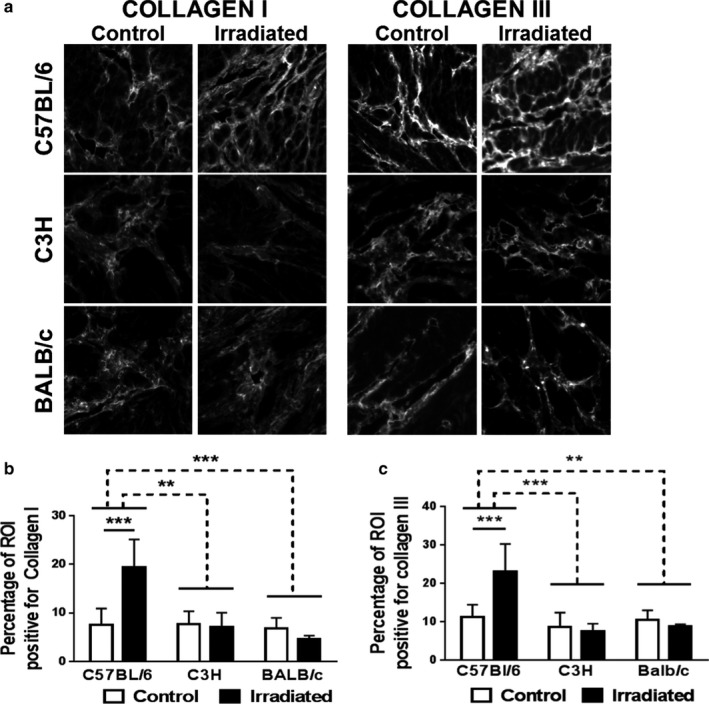
Collagen I and collagen III accumulate in C57BL/6 mice in response to radiation. Bladder strips from irradiated and control mice were stained for collagen I or for collagen III and imaged using fluorescent microscopy. (a) Representative images (100 x 100 µm) of each mouse strain and treatment group are provided for collagen I and collagen III immunofluorescence. (b–c) Percentage of tissue‐positive for collagen I or collagen III staining. Collagen I and III density is significantly elevated in C57BL/6 mice, but not in the C3H or BALB/c strains. Results are mean ± *SD* of *n* = 3–6 mice. Dashed line: ANOVA; Full line: multiple t‐test. **p < .01, *** *p* < .001

**Figure 6:**
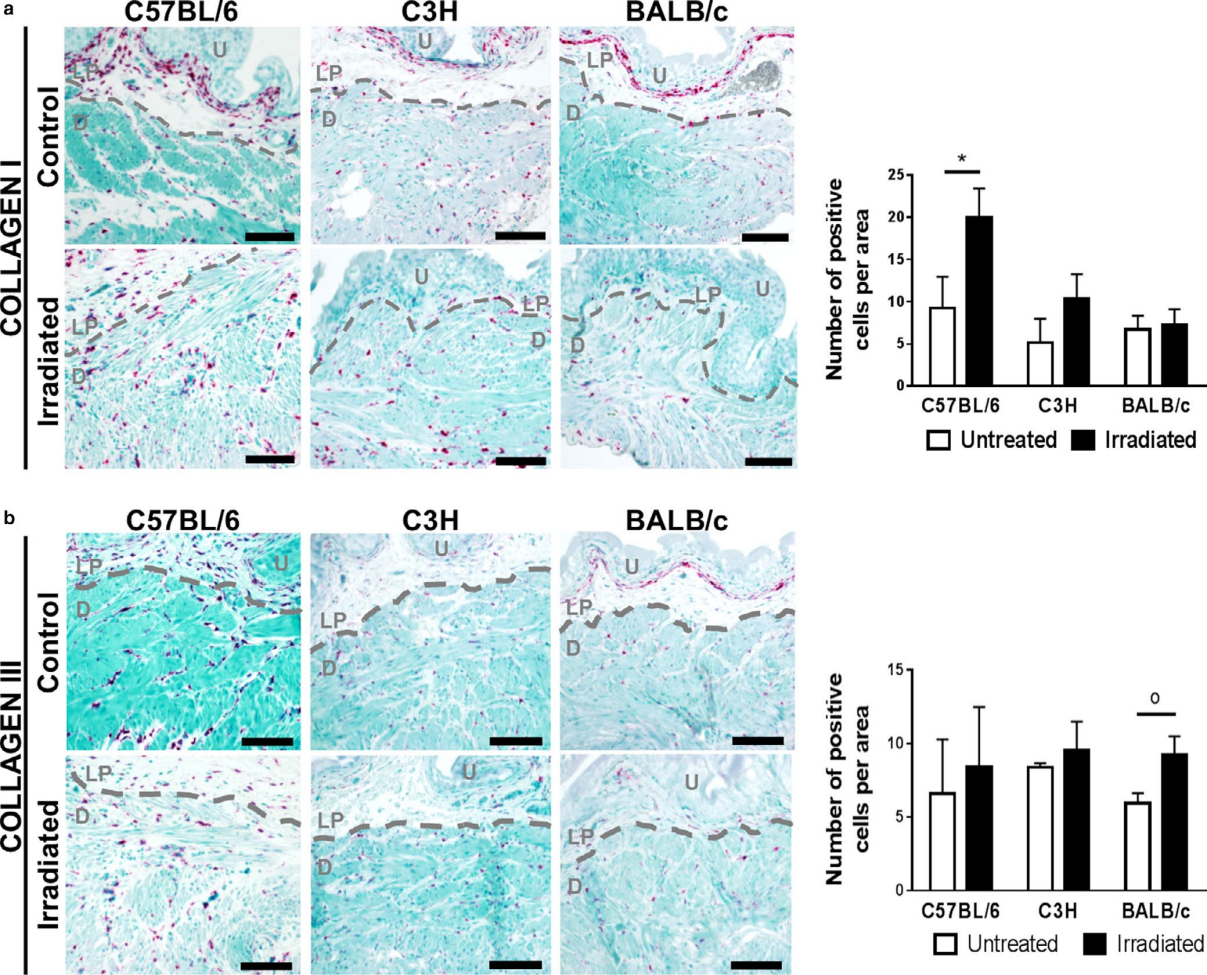
Number of collagen I‐expressing cells is increased in irradiated C57BL/6 detrusor. Bladder strips from irradiated and control mice were stained for collagen I or for collagen III mRNA and counterstained with methyl green. Collagen I and III mRNA (pink) is apparent in both control and irradiated bladder sections in all three mouse strains. The number of collagen I and III‐producing cells in the detrusor is represented in the bar graphs. The number of collagen I‐expressing cells is significantly elevated in C57BL/6 mice after irradiation. Dashed line separates the urothelium (U) and lamina propria (LP) from the detrusor muscle (D). Results are mean ± *SD* of *n* = 3–4 mice. Scale bar = 100 µm. ^o^ *p* ≤ .08, * *p* < .05

We next tested whether radiation increases the amount of collagen I and III, fibrillar collagens that are abundant in fibrotic tissues (Figure 5). Univariate analysis demonstrates a significant interaction between strain and radiation on collagen I (*p* < .001, partial η^2^ = 0.561) and III (*p* = .001, partial η^2^ = 0.512) content in the bladder. The Levene's test showed difference in homogeneity in the data for both collagen I and III. The amount of collagen I and III in C57BL/6 mice is significantly different from C3H and BALB/c mice (Figure 5a–c). Within the C57BL/6 strain, radiation significantly increases collagen I and III immunofluorescence in the bladder (*p* < .001). We subsequently measured abundance of collagen I and III RNA‐positive cells using RNAScope (Figure 6). No interaction between strain and radiation treatment is observed with respect to the number of collagen I and III RNA‐positive cells. However, in C57BL/6 mice, the number of collagen I‐expressing cells is significantly elevated (*p* = .0226) (Figure 6). In addition, radiation noticeably increases cellular staining intensity of collagen I and III RNA (Figure 6). Intensely stained collagen I and III mRNA‐positive cells are common in the lamina propria of C57BL/6 and C3H mice. However, the number of collagen‐producing cells is strongly diminished and at times even absent in the lamina propria of irradiated BALB/c mice versus littermate controls (Figure 6). This appears to coincide with thinning of the lamina propria.

Using multiple linear regression analysis, we found that the number of collagen‐expressing cells (RNAScope) significantly predicts average void spot size (void spot assay) in C57BL/6 mice. When the average spot size is set as the response variable, both the number of collagen I (Beta = −3.882, SE = 0.8399, 95% Wald CI (−5.528, −2.235), *p* < .001) and the number of collagen III (Beta = 4.189, SE = 1.0687, 95% Wald CI (2.094, 6.284), *p* < .001)‐producing cells significantly contribute to predicting the average size per void by the following formula: Average spot size = 60.838 – 3.882 * number of collagen 1 positive cells + 4.189 * number of collagen 3 positive cells (Likelihood Ratio Chi‐square test = 9.831, with *df* = 2 and *p*‐value = 0.007). A similar analysis for the BALB/c mouse strain does not yield a statistically significant result.

**Figure 7:**
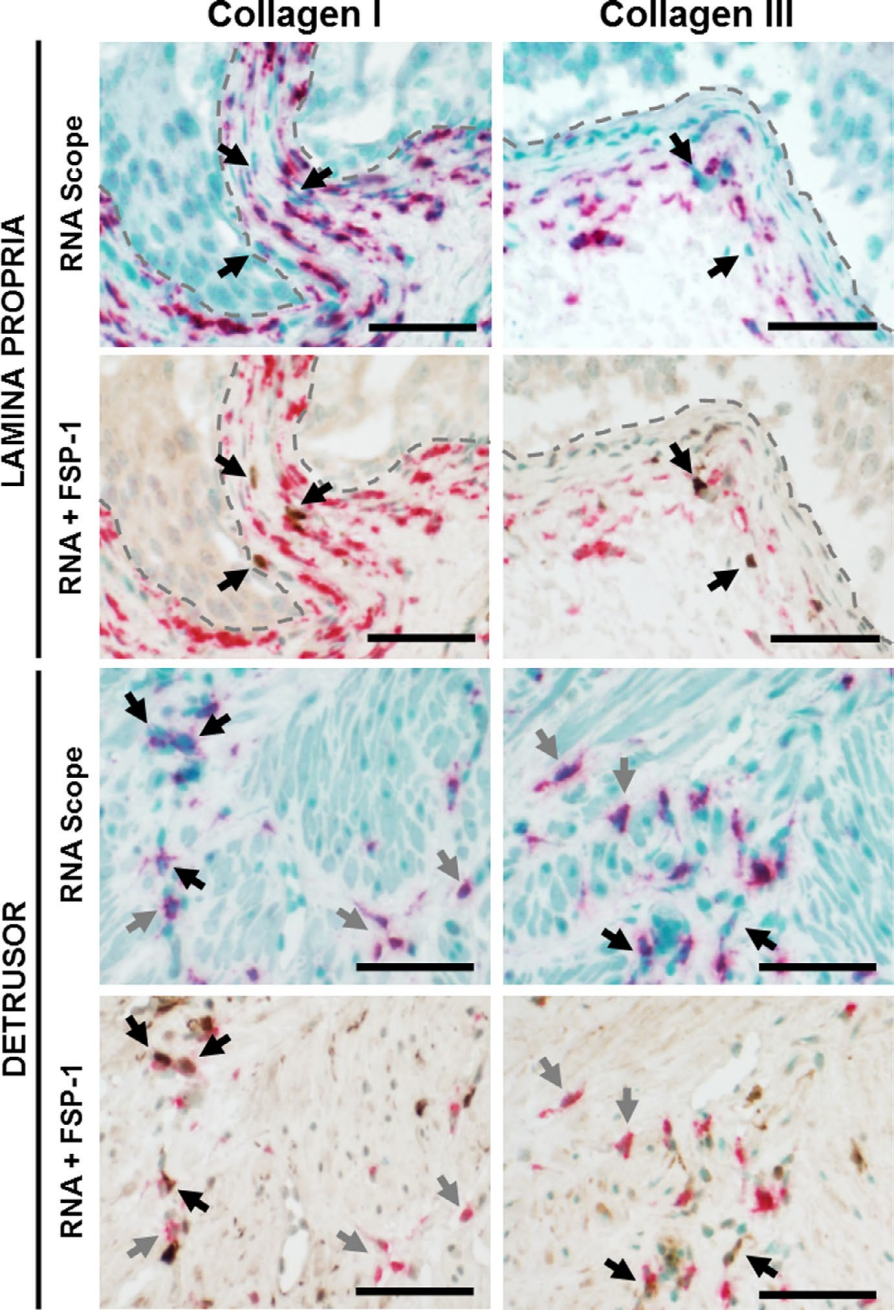
FSP‐1–positive cells only partially responsible for collagen I and III production in irradiated bladders. Bladder strips from irradiated mice were costained for collagen I or for collagen III mRNA (pink) and for fibroblasts (FSP‐1, brown). U: urothelium; LP: lamina propria. Black arrow: FSP‐1 positive cells. Gray arrow: collagen only positive cells. Scale bar = 50 µm

Colocalization staining with fibroblast‐specific protein 1 (FSP‐1) reveals that in the lamina propria of C57BL/6 mice the vast majority of collagen I and III‐producing cells are negative for FSP‐1 (Figure 7 – gray arrows). The few FSP‐1 positive cells in the lamina propria appear to be negative or low expressers of collagen I and III mRNA (Figure 7 – black arrows). In the detrusor, collagen I and III mRNA are expressed by both FSP‐1 positive, as well as FSP‐1 negative cells (Figure 7). In addition, both collagen positive and negative FSP‐1 cells are found within the detrusor.

## 4 DISCUSSION

Radiation is a common cancer therapy (Society AC., 2016). Radiation often induces fibrosis in benign tissues within the radiation field. Factors such as total radiation dose and number of fractions increase a patient's risk of developing RC. However, it still remains a challenge to predict which patients will develop radiation‐induced bladder fibrosis or RC. We compared the anatomical, histological, and physiological responses to radiation across C57BL/6, C3H, and BALB/c female mice. The responses differed across mouse strains, suggesting that genetic background is a determinant of susceptibility to RC. We also show that C57BL/6 mice are highly susceptible to RC, identifying this mouse strain as an appropriate model for future mechanistic studies.

Radiation‐induced fibrosis is mainly studied in lung and intestinal tissue, while fibrosis due to toxic chemicals (bleomycin), obstruction, diet, diabetes, or other external factors are studied in a wider number of organ systems (lung, liver, kidney, heart, and intestine) (Walkin et al., 2013). As was observed in other organs, we find that C57BL/6 mice are prone to fibrosis, C3H mice are resistant, and BALB/c mice have an intermediate phenotype in response to irradiation. Previous studies have examined the effects of radiation on individual mouse strains. Jaal et al. irradiated bladders of female C3H/Neu mice and evaluated bladder tissue at 3, 4, 6, 8, and 12 months post‐radiation (Jaal & Dorr, 2006, 2006, and 2006). They showed that bladder radiation at the early‐stage resulted in an increase of the pro‐inflammatory protein COX2 and an impaired urothelial barrier, and at a later stage caused leaky blood vessels. However, they did not describe fibrosis in their radiation model. Ikeda and colleagues irradiated female C57BL/6 mice and demonstrated a rapid and significant fibrotic response by 6 weeks post‐radiation that coincided with increased micturition frequency (Ikeda et al., 2018). However, the accelerated fibrotic response described by Ikeda and colleagues is likely due to their radiation method; an abdominal incision is made prior to treatment and the bladder is pulled outside of the abdomen during radiation to avoid radiation exposure to surrounding tissue. The previously described bladder radio‐sensitivity of C57BL/6 and C3H mice in response to radiation is consistent with our findings. It is important to note that the strength and uniqueness of our model is the use of the SARRP, which, unlike other studies, more closely mimics the radiation treatment used in a clinical setting and minimizes radiation toxicity or damage to surrounding tissues.

In this study, we show that radiation increases micturition frequency and decreases primary and average individual void size in C57BL/6 and, in a lesser extent, in BALB/c mice. While our methodology was not suitable to measure micturition patterns in C3H mice, previous studies have demonstrated altered micturition frequency in C3H mice after radiation (Lundbeck, Nielsen, & Stewart, 1993; Lundbeck, Oussoren, & Stewart, 1993; Zwaans et al., 2016). These changes are consistent with a functional decrease in bladder capacity. RC patients also have diminished bladder capacity, which is believed to occur as a result of fibrosis (Fry et al., 2018). We used two different histological methods to quantify radiation‐induced fibrosis in the bladder: Masson's trichrome and Picrosirius Red staining. Masson's trichrome staining is a general stain for extracellular matrix which showed a statistically significant increase in fibrotic tissue in response to radiation in C57BL/6, but not in C3H or BALB/c mice. The ANOVA test showed a significant interaction effect between strain and radiation treatment, suggesting that the effect of radiation on fibrosis, as measured by Masson's trichrome staining, is at least in part explained by strain differences. However, a Bonferroni pairwise comparison test failed to yield a significant response, possibly due to the lack of homogeneity in the data (Levene's test *p* = .004) or a low *n*‐value. Picrosirius Red staining, which identifies only total collagen, demonstrated that the effect of radiation on total collagen is dependent on strain. Radiation treatment induced a significant increase in collagen density in the C57BL/6 and BALB/c strains, but not in the C3H mice. Thus radiation may not increase total extracellular matrix in BALB/c mice (Masson's Trichrome), but instead alters the composition of the extracellular matrix. This altered composition could be responsible for the functional bladder changes observed in the micturition assays. No changes in collagen I or III were observed in BALB/c mice, meaning that other collagen subtypes, for example, fibrillary collagens 2 and 5, are likely responsible for the increase in total collagen after radiation. This change in extracellular matrix composition might also explain the modest change in collagen fiber length observed in the BALB/c mice. The lack of fibrosis in C3H mice is consistent with findings from other groups (Iwakawa et al., (2004); Johnston et al., 1995; Johnston et al., 1996; Skwarchuk & Travis, 1998). Varying expression of numerous proteins, including collagen I, fibronectin, various cytokines, TNF‐α, and others have been proposed to be responsible for the difference in fibrotic response after radiation in C57BL/6 versus C3H mice (Iwakawa et al., 2004; Johnston et al., 1995, 1996; Skwarchuk & Travis, 1998).

At study endpoint, macroscopic bladder images demonstrated altered bladder morphology in all three mouse strains. The change in bladder shape can be explained in C57BL/6 mice and BALB/c mice by increased fibrosis/total collagen or altered extracellular matrix composition, and was supported by voiding dysfunction. We did not observe any changes in fibrosis in the C3H strain, which is consistent with the findings of other studies (Lundbeck, Nielsen, et al., 1993; Lundbeck, Oussoren, et al., 1993). It is noteworthy that these previous studies did observe functional bladder changes in response to irradiation. Therefore, tissue alterations, other than fibrosis, possibly contribute to the altered morphology such as bladder stiffness, change in extracellular matrix composition, or reduced muscle contractility. We did observe, a reduction in collagen I and III production in the lamina propria in response to irradiation, predominantly in the BALB/c mice and in lesser extent in the C3H mice. This coincided with a notable thinning of the lamina propria. This histological change might contribute to voiding dysfunction and/or the macroscopic changes in the bladder after irradiation.

The increased total collagen in C57BL/6 mice is, at least in part, due to increased collagen I and III deposition. RNAScope analysis demonstrated that both collagens are still actively being transcribed at 12 months post‐irradiation. The number of collagen I‐producing cells was significantly increased at 12 months after radiation in C57BL/6 mice. This indicates that radiation‐induced bladder fibrosis is a chronic condition with a long latency. Notably, age‐induced voiding changes have been previously described in C57BL/6 mice by 18 months of age, thus at 12 months post‐radiation (14 months of age) age could start contributing to elevated collagen production (Yu et al., 2014). Previous studies have shown that activated fibroblasts and myofibroblasts are the most abundant producers of collagens type I and III in fibrosis (Karsdal et al., 2017). However, our RNAScope staining in conjunction with the fibroblast marker FSP‐1 revealed that fibroblasts are not the only producers of collagen I and III in the bladder. One possible explanation is that radiation stimulates collagen production from a unique cell type. Alternatively, FSP‐1 positive cells may be responsible for collagen deposition in the earlier stages of radiation‐induced bladder fibrosis. This hypothesis will be investigated in future studies.

Multiple linear regression analysis demonstrated that the number of collagen I and collagen III‐producing cells can predict the average voided volume, an indicator of bladder compliance, without taking treatment into consideration. The regression model we obtained would suggest an inverse association between the number of collagen type I‐producing cells and the average voided volume, while an increase in the number of collagen type III‐expressing cells would be associated with an increase in average voided volume at 12 months post‐radiation. Both collagens are essential in the creation of a stable scaffold during the initial stages of wound healing and in the development of mild‐to‐moderate fibrosis. However, advancing from moderate‐to‐severe fibrosis is associated with a relative increase in collagen type I over collagen type III (Karsdal et al., 2017). Thus, our linear regression model would suggest that the C57BL/6 mice are in the stages of developing more advanced fibrosis, hereby contributing to bladder dysfunction.

There were some additional observations in this study that are worth noting. First, the C3H mouse strain was destructive to the filter paper during void spot assay and therefore are not a good strain for the methodology used here. In a previous study, we measured micturition frequency in C3H mice post‐irradiation using a pH sensitive paper covered by a metal grid for an overnight study (16 hr) during which mice had unlimited access to food and water (Zwaans et al., 2016). Here, we did see an increase in micturition frequency starting at 17 weeks post‐irradiation. However, the increase in number of voids did not always correspond to a change in void size. The disadvantage of the pH sensitive paper is poor absorption of the urine in comparison to filter paper. Thus, if using C3H mice, filter paper covered with a metal grid to avoid destruction of the paper might be a more preferable approach (Hill, Zeidel, Bjorling, & Vezina, 2018).

Second, throughout the study we lost five mice prior to the study endpoint: Three irradiated C57BL/6 mice, one control C3H mouse, and one irradiated BALB/c mouse. Two of the C57BL/6 mice had severe voiding dysfunction and a hunched posture prior to death, suggesting that irradiation‐induced bladder damage may be in part responsible for their death. The third C57BL/6 mouse was euthanized due to a skin lesion (ulcerative dermatitis) far outside the radiation field that would not heal despite veterinary intervention. The irradiated BALB/c mouse was euthanized shortly before the end of the study due to severe weight loss with unknown cause.

Based on this study, we recommend the use of female C57BL/6 mice to study radiation‐induced bladder fibrosis. First, they develop a clear fibrotic response that coincides with voiding dysfunction and increased collagen I and III. Second, female C57BL/6 mouse bladders are easy to catheterize and instill, which is advantageous when considering instillation of potential therapies. C57BL/6 mice at times do chew the filter paper, but we were able to minimize this by adding some enrichments to the cage (e.g., rounded house with open bottom and small piece of string paper). Finally, many transgenic mouse models are available on a C57BL/6 background that could be used to elucidate underlying mechanism in future studies. C3H mice represent a good model for understanding resistance to developing fibrosis in response to radiation. Together with a fibrosis prone model, the C3H mice would be a great resource to help identify the genetic locus responsible for radiation‐induced bladder fibrosis.

## 5 CONCLUSION

In this study, we investigated the effect of pelvic radiation on bladder health in three commonly used mouse strains: C57BL/6, C3H, and BALB/c. We found that C57BL/6 mice are most prone to developing fibrosis in the bladder in response to irradiation, which is supported by alterations in bladder micturition including increased voiding frequency and smaller void size. Thus, genetic variation may also contribute to which patients develop RC in response to irradiation treatment, which could impact treatment approach and follow‐up recommendations. Increased fibrosis in C57BL/6 mice is, at least in part, attributed to elevated deposition of collagen I and collagen III. However, at 1‐year post‐irradiation, collagen is produced by other cells in addition to fibroblasts. Together, this data demonstrates that different mouse strains have different bladder radio‐sensitivity, and that cell types other than fibroblasts are responsible for collagen production in the development of radiation cystitis.

## CONFLICT OF INTERESTS

All authors declare no competing interests.
