# Supplementary figures and images for: Radiation cystitis modeling: A comparative study of bladder fibrosis radio‐sensitivity in C57BL/6, C3H, and BALB/c mice
Source: Physiol Rep. 2020 Feb 28;8(4):e14377. doi: 10.14814/phy2.14377 (PMC7048381; doi:10.14814/phy2.14377)

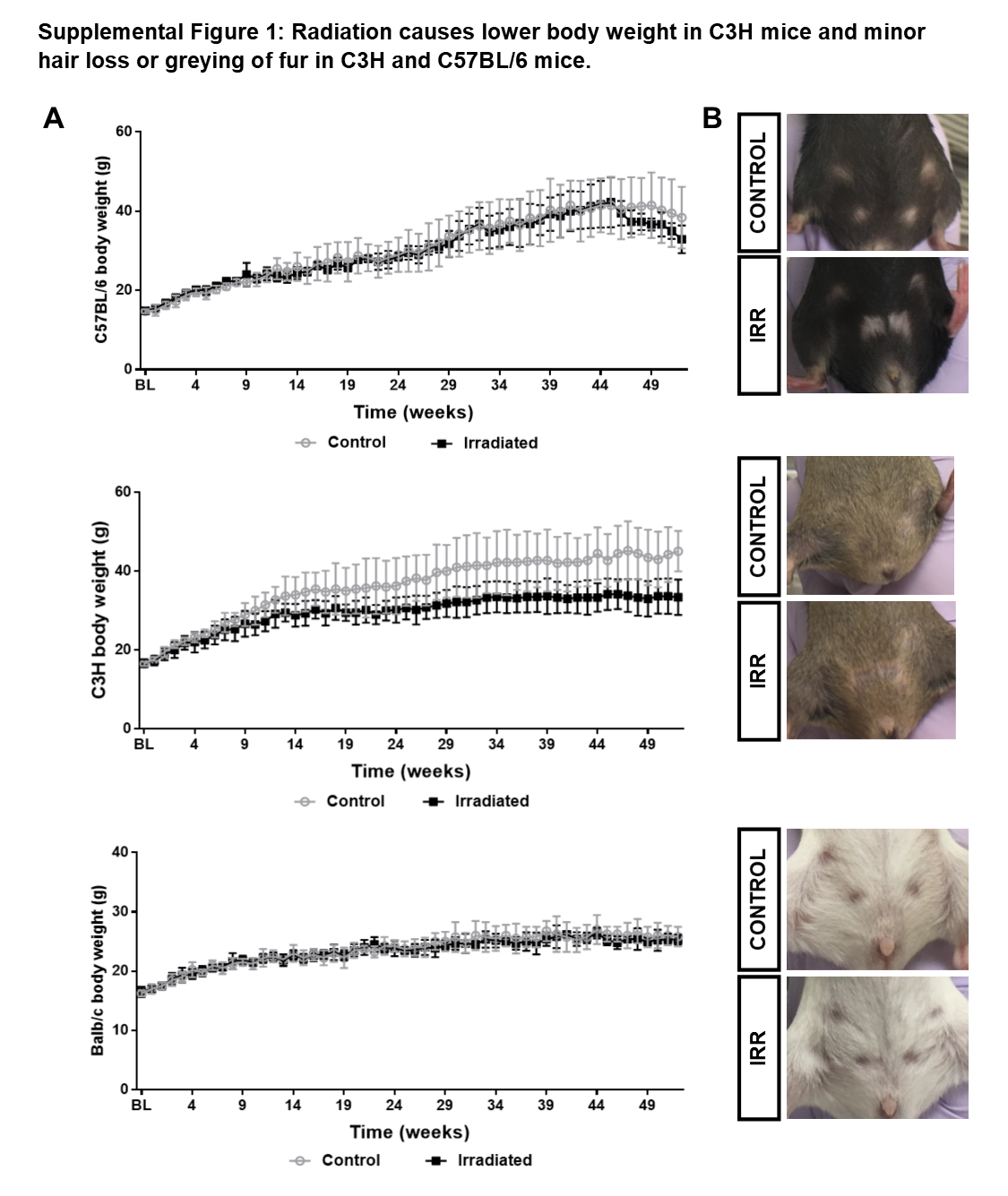

Supplement: Supplementary file 1 [file PHY2-8-e14377-s001.tif]
